# Variability of serum IgG sialylation and galactosylation degree in women with advanced endometriosis

**DOI:** 10.1038/s41598-021-85200-x

**Published:** 2021-03-10

**Authors:** Katarzyna Sołkiewicz, Hubert Krotkiewski, Marcin Jędryka, Ewa M. Kratz

**Affiliations:** 1 Department of Laboratory Diagnostics, Division of Laboratory Diagnostics, Faculty of Pharmacy, Wroclaw Medical University, Borowska Street 211A, 50-556 Wrocław, Poland; 2 Hirszfeld Institute of Immunology and Experimental Therapy, Polish Academy of Sciences, Weigla Street 12, 53-114 Wrocław, Poland; 3 Department of Oncology, Gynecological Oncology Clinic, Faculty of Medicine, Wroclaw Medical University, Hirszfeld Square 12, 53-413 Wrocław, Poland; 4 Department of Oncological Gynecology, Wroclaw Comprehensive Cancer Center, Hirszfeld Square 12, 53-413 Wrocław, Poland

**Keywords:** Biochemistry, Immunology, Biomarkers, Diseases

## Abstract

Endometriosis is an inflammatory disease which diagnostics is difficult and often invasive, therefore non-invasive diagnostics methods and parameters are needed for endometriosis detection. The aim of our study was to analyse the glycosylation of native serum IgG and IgG isolated from sera of women classified as: with endometriosis, without endometriosis but with some benign ginecological disease, and control group of healthy women, in context of its utility for differentiation of advanced endometriosis from the group of healthy women. IgG sialylation and galactosylation/agalactosylation degree was determined using specific lectins: MAA and SNA detecting sialic acid α2,3- and α2,6-linked, respectively, RCA-I and GSL-II specific to terminal Gal and terminal GlcNAc, respectively. The results of ROC and cluster analysis showed that the serum IgG MAA-reactivity, sialylation and agalactosylation factor may be used as supplementary parameters for endometriosis diagnostics and could be taken into account as a useful clinical tool to elucidate women with high risk of endometriosis development. Additionally, we have shown that the analysis of native serum IgG glycosylation, without the prior time-consuming and expensive isolation of the protein, is sufficient to differentiation endometriosis from a group of healthy women.

## Introduction

Endometriosis, defined as the presence of endometrial glands and stroma like lesions outside of the uterus^1^, has been diagnosed in 176 million women worldwide^2^. The disease has a significant impact on women fertility, as it occurs in more than one-third of infertile women and two-thirds of women with chronic pelvic pain^3^. Endometriosis, although not a malignant disease, invades surrounding organs^4^ and the lesions can appear as ovarian cyst, peritoneal or superficial implants, or deep infiltrating disease, which induces a chronic, inflammatory reaction^2^. According to the revised American Fertility Society (rAFS) classification^5^ and up-to-dated by American Society for Reproductive Medicine (ASRM)^6^, based on severity and anatomical extension of changes, endometriosis is graded into four stages: minimal, mild, moderate and severe (I, II, III and IV stage, respectively). The etiology of this disease remains obscure despite several hypotheses how endometriosis develop. Therefore, many various factors, hormonal, inflammatory or immunological, may determine whether deposits in the pelvic cavity may implant and persist^7–9^. Endometriosis seems to be associated with antibody self-reactivity and chronic local inflammation, coexisting also with autoimmune disease, but this does not seem to be a common phenomenon^10^. The presence of autoreactive antibodies in serum of some endometriosis women may be a natural by-product of inflammation and local tissue destruction. The finding of antibodies to endometrial and ovarian nuclear antigens in women with endometriosis supports the hypothesis of endometriosis being a multiple antibody autoimmune condition^10^. The ‘gold diagnostic standard’ for endometriosis identification is laparoscopic inspection with histologic confirmation after biopsy, however, the correlation between clinical symptoms and disease stage is poor, and invasive testing carries required anaesthesia and surgical skills and produces potential risk of complications^11,12^. Many studies focus on searching for biochemical markers with high sensitivity and specificity for non-invasive diagnostics or screening of endometriosis^11–13^ as well as aim better understanding molecular mechanisms responsible for disease development^14–16^. Proper identification of endometriosis is still problematic in gynaecologic practice, therefore extensive research for new biomarkers give hope for more accurate diagnostics, especially in the advanced stages of the disease.

Glycosylation, one of the most common post-translational modifications of secretory and membrane proteins, plays an important role in biological processes, such as cell recognition and adhesion, cell–cell communication and cell–cell interactions^17^, and plays a key role in antibodies function^18^. Immunoglobulin G (IgG), 150-kDa glycoprotein, is the most abundant immunoglobulin in blood (represents about 75% of serum immunoglobulins), involved in the pathogenesis and progression of many diseases. IgG plays a key role in defending the host against microbial infections, but also in pathological events (*inter alia* inflammation) being a cause of various diseases. Immunoglobulin G can activate a variety of effector mechanisms, such as complement-dependent cytotoxicity, antibody-dependent cellular cytotoxicity and phagocytosis^19,20^. IgG glycans are essential for the proper activity of the immune system. The presence or absence of one sugar moiety in oligosaccharide structure of N-glycan may result in the stimulation or suppression of immune response. The contribution of IgG Fc glycosylation changes was documented for the pathogenesis of rheumatoid arthritis, Crohn’s disease and lupus erythematosus. Found in these diseases the decreased IgG galactosylation and sialylation, activates effector cells and initiates an inflammatory response^18^. IgG has one biantennary N-linked glycan attached to asparagine 297^14,21^, which consists of a constant heptameric core structure containing three mannose residues and four GlcNAc residues and may contain additional core fucose, as well as bisecting GlcNAc. Additionally, the branching arms (α-6 and α-3) may have variable glycosylation pattern consisting of terminal galactose and sialic acids^14,22,23^. The profile and degree of IgG glycosylation can vary in various pathological conditions and its glycans composition can alter the effector functions of immunoglobulin by modulating its affinity for ligands, such as Fcγ receptors (FcγRs)^19^. Many reports described variations of IgG glycosylation, especially the degree of glycosylation, related to age, sex, heritability and pregnancy, as well as to autoimmune diseases and cancers^24–30^. It is already documented, that in rheumatoid arthritis patients, serum IgG galactosylation of its conservative N-glycans (Asn-297) in CH_2_ domains of the heavy chains is decreased, and that IgG agalactosylation is proportional to disease severity^31^.

In the present study we were interested, if the expression of serum IgG sialylated and galactosylated/agalactosylated glycoforms is characteristic for advanced stages of endometriosis. Another important aspect was the comparison of native serum IgG glycosylation with glycosylation of IgG isolated from serum, and evaluation the potential diagnostic suitability of glycan analysis when glycoprotein is not isolated earlier from biological fluid. The degree of IgG sialylation and galactosylation/agalactosylation was analysed using a modified solid phase enzyme-linked immunosorbent assay, lectin-ELISA^31–34^.

## Results

**Figure 1:**
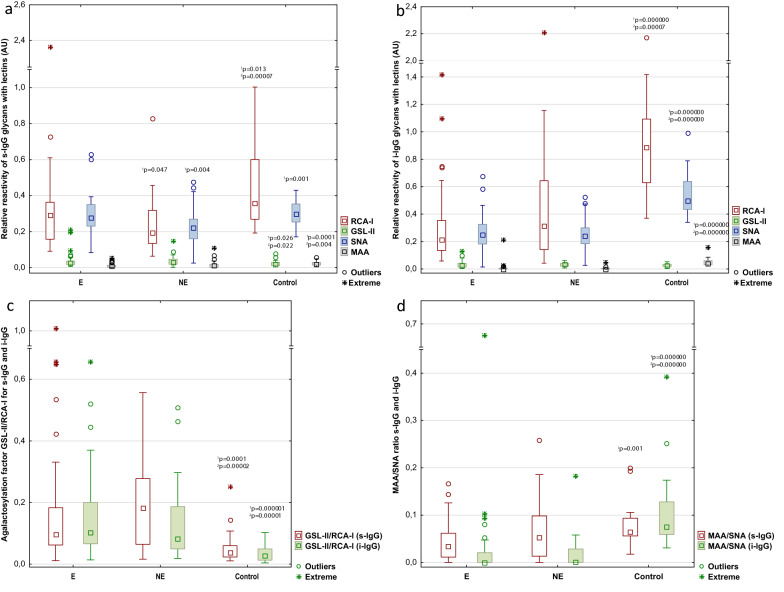
Relative reactivities of serum native IgG (s-IgG)—(**a**), and isolated serum IgG (i-IgG)—(**b**) glycans with specific lectins. GSL-II: *Griffonia simplicifolia* lectin II, recognizing terminal GlcNAc; RCA-I: *Ricinus communis* agglutinin I, recognizing terminal Gal; MAA: *Maackia amurensis* agglutinin, recognizing sialic acid α2,3-linked; SNA: *Sambucus nigra* agglutinin, recognizing sialic acid α2,6-linked. E—endometriosis, NE—no endometriosis and Control—group of healthy women. The relative reactivities with lectins were expressed in absorbance units (AU). (**c**)—the values of agalactosylation factor GSL-II/RCA-I for IgG glycans. (**d**)—the values of MAA/SNA ratio for IgG glycans. MAA: *Maackia amurensis* agglutinin, recognizing sialic acid α2,3-linked; SNA: *Sambucus nigra* agglutinin, recognizing sialic acid α2,6-linked. Significant differences versus groups: ^1^E, ^2^NE. Median is indicated as square. A two-tailed *p*-value of less than 0.05 was considered as significant.

The relative reactivities of serum IgG glycans with selected panel of biotinylated lectins, specific to sialic acid (MAA, SNA), terminal Gal (RCA-I) and terminal GlcNAc (GSL-II) are presented in Table 1 as mean values of absorbances and standard deviations (SD) for each analysed group. The distributions and median values of IgG relative reactivities with lectins tested, measured for the control, E and NE groups, are presented in Fig. 1. The additional Tables and Figures are presented in Supplementary materials (Tables 1S, 2S, 3S and Figs. 1S, 2S, 3S, 4S, 5S).

**Table 1:** Relative reactivity of s-IgG and i-IgG glycans with specific lectins.

Groups	Relative reactivity with lectins (AU)											
	MAA (s)	MAA (i)	SNA (s)	SNA (i)	MAA/SNA ratio (s)	MAA/SNA ratio (i)	GSL-II (s)	GSL-II (i)	RCA-I (s)	RCA-I (i)	GSL-II /RCA-I ratio (s)	GSL-II /RCA-I ratio (i)
E n = 40	0.011 ± 0.012	0.009 ± 0.031	0.288 ± 0.105	0.263 ± 0.144	0.042 ± 0.048	0.030 ± 0.099	0.037 ± 0.041	0.034 ± 0.028	0.331 ± 0.343	0.341 ± 0.325	0.169 ± 0.200	0.145 ± 0.132
NE n = 36	0.016 ± 0.021	0.004 ± 0.009	0.227 ± 0.098	0.250 ± 0.115	0.063 ± 0.060	0.018 ± 0.034	0.036 ± 0.028	0.032 ± 0.014	0.232 ± 0.147	0.448 ± 0.443	0.197 ± 0.147	0.135 ± 0.120
			^1^*p* = 0.004						^1^*p* = 0.047			
Control n = 19	0.023 ± 0.013	0.051 ± 0.032	0.301 ± 0.071	0.546 ± 0.165	0.081 ± 0.048	0.107 ± 0.087	0.023 ± 0.018	0.025 ± 0.013	0.468 ± 0.259	0.925 ± 0.419	0.054 ± 0.058	0.035 ± 0.028
	^1^*p* = 0.0001	^1^*p* = 0.000000		^1^*p* = 0.000000	^1^*p* = 0.001	^1^*p* = 0.000000	^1^*p* = 0.026		^1^*p* = 0.013	^1^*p* = 0.000000	^1^*p* = 0.0001	^1^*p* = 0.000001
	^2^*p* = 0.004	^2^*p* = 0.000000	^2^*p* = 0.001	^2^*p* = 0.000000		^2^*p* = 0.000000	^2^*p* = 0.022		^2^*p* = 0.00007	^2^*p* = 0.00007	^2^*p* = 0.00002	^2^*p* = 0.00001

The results are expressed in absorbance units (AU) as mean values ± SD. Serum IgG glycan reactivities with lectins were estimated by direct lectin-ELISA and expressed in absorbance units (AU). MAA: *Maackia amurensis* agglutinin, recognizing sialic acid α2,3-linked; SNA: *Sambucus nigra* agglutinin, recognizing sialic acid α2,6-linked; GSL-II: *Griffonia simplicifolia* lectin II, recognizing terminal GlcNAc; RCA-I: *Ricinus communis* agglutinin I, recognizing terminal Gal. Significant differences versus groups: ^1^with endometriosis (E), ^2^non-endometriosis (NE). Control—group of healthy women. s-IgG—serum native IgG; i-IgG—serum IgG isolates. Statistically significant differences were accepted for a *p-*value of less than 0.05.

**Table 2:** The correlations between relative reactivities of IgG glycans with lectins.

Correlations between lectins relative reactivity	s-IgG	i-IgG
	Spearman rank coefficient (*r*)	Spearman rank coefficient (*r*)
RCA-I vs. GSL-II/RCA-I	*r* = − 0.679 *p* = 0.000000	*r* = − 0.770 *p* = 0.000000
RCA-I vs. SNA	*r* = 0.743 *p* = 0.000000	*r* = 0.781 *p* = 0.000000
RCA-I vs. MAA		*r* = 0.509 *p* = 0.000000
RCA-I vs. MAA/SNA		*r* = 0.465 *p* = 0.000000
GSL-II vs. GSL-II/RCA-I	*r* = 0.770 *p* = 0.000000	*r* = 0.389 *p* = 0.00006
GSL-II vs. MAA	*r* = 0.357 *p* = 0.0002	
GSL-II vs. MAA/SNA	*r* = 0.364 *p* = 0.0002	
SNA vs. MAA		*r* = 0.538 *p* = 0.000000
SNA vs. MAA/SNA		*r* = 0.484 *p* = 0.000000
SNA vs. GSL-II/RCA-I	*r* = − 0.408 *p* = 0.000000	*r* = − 0.588 *p* = 0.000000
MAA vs. MAA/SNA	*r* = 0.927 *p* = 0.000000	*r* = 0.978 *p* = 0.000000
MAA vs. GSL-II/RCA-I		*r* = − 0.301 *p* = 0.000000

Serum native IgG—s-IgG; serum IgG isolates—i-IgG. MAA: *Maackia amurensis* agglutinin, recognizing sialic acid α2,3-linked; SNA: *Sambucus nigra* agglutinin, recognizing sialic acid α2,6-linked; GSL-II: *Griffonia simplicifolia* lectin II, recognizing terminal GlcNAc; RCA-I: *Ricinus communis* agglutinin I, recognizing terminal Gal. A two-tailed *p*-value of less than 0.05 was considered as significant.

**Table 3:** The correlations between relative reactivities of s-IgG and i-IgG glycans with lectins.

Correlations between lectins relative reactivity s-IgG vs. i-IgG	Spearman rank coefficient (*r*)
RCA-I vs. RCA-I	*r* = 0.226 *p* = 0.02
GLS-II vs. GLS-II	*r* = 0.440 *p* = 0.000005
GSL-II/RCA-I vs. GSL-II/RCA-I	*r* = 0.365 *p* = 0.0002
SNA vs. SNA	*r* = 0.421 *p* = 0.00001
MAA vs. MAA	*r* = 0.487 *p* = 0.000000
MAA/SNA vs. MAA/SNA	*r* = 0.449 *p* = 0.000003

Serum native IgG—s-IgG; serum IgG isolates—i-IgG. GSL-II: *Griffonia simplicifolia* lectin II, recognizing terminal GlcNAc; RCA-I: *Ricinus communis* agglutinin I, recognizing terminal Gal; MAA: *Maackia amurensis* agglutinin, recognizing sialic acid α2,3-linked; SNA: *Sambucus nigra* agglutinin, recognizing sialic acid α2,6-linked. A two-tailed *p*-value of less than 0.05 was considered as significant.

### Relative reactivity of IgG with MAA and SNA

The expression of MAA-reactive α2,3-linked sialic acid in s-IgG was significantly lower in E and NE groups (0.011 ± 0.012 AU, median 0.007 AU, *p* = 0.0001 and 0.016 ± 0.021 AU, median 0.012 AU, *p* = 0.004, respectively) than for the control group (0.023 ± 0.013 AU, median 0.020 AU). Also the relative reactivities of i-IgG with MAA were higher for control group (0.051 ± 0.0032 AU, median 0.037 AU) than in E and NE groups (0.009 ± 0.031 AU, median 0.000 AU, *p* = 0.000000 and 0.004 ± 0.009 AU, median 0.000 AU, *p* = 0.000000, respectively).

The relative reactivities of s-IgG with SNA-reactive sialic acid α2,6-linked were significantly lower in NE group (0.227 ± 0.098 AU, median 0.222 AU) than those measured for the control group (0.301 ± 0.071 AU, median 0.298 AU, *p* = 0.001) and endometriosis (0.288 ± 0.105 AU, median 0.278 AU, *p* = 0.004) groups. Relative reactivities of i-IgG with SNA were significantly higher for control group (0.546 ± 0.165 AU, median 0.496 AU) than for E (0.263 ± 0.144 AU, median 0.250 AU, *p* = 0.000000) and NE (0.250 ± 0.115 AU, median 0.240 AU, *p* = 0.000000) groups. The ratio of MAA/SNA relative reactivities was significantly higher in control group (0.081 ± 0.048, median 0.064 for s-IgG and 0.107 ± 0.087, median 0.075, for i-IgG) than in E (0.042 ± 0.048, median 0.034, *p* = 0.001 and 0.030 ± 0.099, median 0.000, *p* = 0.000000, respectively) and NE groups (0.018 ± 0.034, median 0.001, *p* = 0.000000 for i-IgG only).

### Relative reactivity of IgG with GSL-II and RCA-I

As shown in Table 1, the relative reactivities of s-IgG with GlcNAc-specific lectin GSL-II in the E (0.037 ± 0.041 AU, median 0.026 AU) and NE (0.036 ± 0.028 AU, median 0.030 AU) groups were significantly higher (*p* = 0.026 and *p* = 0.022, respectively) than those observed for the control group (0.023 ± 0.018 AU, median 0.020 AU). However, there were no significant differences between analysed groups in relative reactivities of i-IgG with GSL-II (*p* > 0.05).

The relative reactivities of s-IgG glycans with galactose-specific lectin RCA-I in the E and NE groups were significantly lower (0.331 ± 0.343 AU, median 0.292 AU, *p* = 0.013 and 0.232 ± 0.147 AU, median 0.195 AU, *p* = 0.00007, respectively) than those observed for the control group (0.468 ± 0.259 AU, median 0.358 AU). Also the expression of RCA-I-reactive IgG glycans was significantly lower in NE than E group (*p* = 0.047). The relative reactivities of i-IgG with galactose-specific RCA-I were significantly lower in E (0.341 ± 0.325 AU, median 0.221 AU, *p* = 0.000000) and NE (0.448 ± 0.443 AU, median 0.315 AU, *p* = 0.00007) groups than those measured for samples from the control group (0.925 ± 0.419 AU, median 0.887 AU). The agalactosylation factor (AF), measured as ratio of GSL-II/RCA-I relative reactivities, was significantly lower in control group (0.054 ± 0.058, median 0.038, for s-IgG and 0.035 ± 0.028, median 0.026, for i-IgG) than in E (0.169 ± 0.200, median 0.088, *p* = 0.0001 and 0.145 ± 0.132, median 0.099, *p* = 0.000001, respectively) and NE groups (0.197 ± 0.147, median 0.182, *p* = 0.00002 and 0.135 ± 0.120, median 0.083, *p* = 0.00001, respectively).

The Spearman rank correlations, analysed between relative reactivities of lectins used with IgG glycans (s-IgG and i-IgG) are shown in Table 2 and Fig. 1S. It was a positive high correlation between relative reactivities of RCA-I and SNA (*r* = 0.743, *p* = 0.00; *r* = 0.781, *p* = 0.00) with glycans of s-IgG and i-IgG. For s-IgG a positive high correlation was observed between GSL-II relative reactivity and AF GSL-II/RCA-I (*r* = 0.770; *p* = 0.00), while a negative high correlation was observed between RCA-I relative reactivity and the AF GSL-II/RCA-I (*r* =  − 0.679; *p* = 0.00), which were also observed for i-IgG *(r* =  − 0.770; *p* = 0.00). For i-IgG a high positive correlations exist between relative reactivities with RCA-I versus MAA and with SNA versus MAA (*r* = 0.509, *p* = 0.00 and *r* = 0.538, *p* = 0.00, respectively). Full correlations were found for s-IgG and i-IgG between MAA relative reactivities and ratio MAA/SNA (*r* = 0.927, *p* = 0.00 and *r* = 0.978, *p* = 0.00, respectively). The correlations between relative reactivities of s-IgG and i-IgG glycans with lectins are presented in Table 3 and Fig. 2S. An average positive correlation in MAA reactivities was observed between glycans of s-IgG and i-IgG (*r* = 0.487, *p* = 0.00).

### ROC curve analysis

The results of ROC curve analysis are showed in Fig. 3S and Table 1S. ROC curve analysis of s-IgG glycans relative reactivities with lectins, and their reactivities ratio, in endometriosis and control group, identified parameters with a sensitivity and specificity of: MAA 0.795, 0.795 (AUC 0.812—moderate clinical value); SNA 0.364, 0.842 (AUC 0.575—limited clinical value); MAA/SNA 0.659, 0.895 (AUC 0.762—moderate clinical value); RCA-I 0.864, 0.474 (AUC 0.699—limited clinical value); GSL-II 0.866, 0.474 (AUC 0.675—limited clinical value); GSL-II/RCA-I 0.750, 0.842 (AUC 0.800—moderate clinical value). For i-IgG the obtained results were following: MAA 0.886, 1 (AUC 0.970—high clinical value); SNA 0.864, 0.947 (AUC 0.943—high clinical value); MAA/SNA ratio 0.773, 1 (AUC 0.916—high clinical value); RCA-I 0.795, 1 (AUC 0.922—high clinical value); GSL-II 0.455, 0.737 (AUC 0.539—limited clinical value); GSL-II/RCA-I ratio 0.864, 0.842 (AUC 0.886—moderate clinical value). For the determination of cut-off points, the Youden index method was used. The clinical value of a laboratory test with AUC can be defined as: 0–0.5—zero, 0.5–0.7—limited, 0.7–0.9—moderate and > 0.9 high^35^.

### Cluster analysis

The usefulness of s-IgG and i-IgG glycans expression for differentiation of endometriosis group from group of healthy women, the relative reactivity with MAA as well as MAA/SNA and GSL-II/RCA ratio were selected from the panel of parameters examined, taking into account the results of ROC analysis (AUC ≥ 0.762; Figs. 4S, 5S and Tables 2S, 3S). The analysis was performed for 59 samples, for which all selected parameters were determined. The analysis for s-IgG and i-IgG was done separately. In case of s-IgG, the first cluster could be distinguished as a homogenous group of 3 endometriosis samples (Fig. 4S, Table 2S) at 100% distance. The next group could be separated at 33% distance (Cluster 2) consists of 12 samples, including 16% of women from whole healthy group. Next cluster (Cluster 3 at 16% distance) comprised 23 subjects, 14 of which were healthy women from the control group (74% of the whole group) and 9 samples belonged to endometriosis patients (22.5% of the whole endometriosis group). Last cluster (Cluster 4) include 21 samples, 19 samples form endometriosis group (47.5% of the whole group).

In cluster analysis of i-IgG (Fig. 5S, Table 3S) first cluster could be separated at distance 92%. This homogenous group consists of 2 endometriosis samples. The next homogenous group could be separated at 69% distance (Cluster 2) in which 4 samples from the control group were gathered. Cluster 3 (homogenous, composed of endometriosis samples) and cluster 4 (gathered 3 control samples and 24 endometriosis samples) were distinguished at distance 40% and 24%, respectively. Cluster 5 include 13 samples, 12 from the control group, which make up 63% of the whole group of healthy women.

## Discussion

This is the first study, to our best knowledge, in which the serum IgG sialylation degree and agalacto-glycans expression was analysed in context of advanced endometriosis. We showed that for native and isolated serum IgG the significantly decreased galactose expression, α2,3-sialylation and value of MAA/SNA ratio, as well as increased expression of agalacto-glycans (for s-IgG only) and level of agalactosylation factor GSL-II/RCA-I in patients with non-endometriosis (for MAA/SNA ratio only for i-IgG) and advanced endometriosis (not for s-IgG SNA-reactivity) in comparison to the healthy women were observed. However, for s-IgG in NE group significantly lower SNA-reactivity and galactose expression, and significantly higher agalactosylation factor GSL-II/RCA-I in comparison to E group were observed. Reduced sialylation and galactosylation is typical for inflammatory condition and abnormal glycosylation patterns of glycoproteins are associated with various diseases, such as inflammation or cancer, what was reported in many independent studies^24,36,37^. Also endometriosis is a disease accompanied by the development of inflammation^10,38^. Presence or absence in IgG glycan structure of distinct residues such as sialic acid and galactose, can alter pro- and anti-inflammatory IgG activities^20^. An absence of sialic acids and low degree of galactosylation might be linked with pro-inflammatory properties of IgG, by facilitating the formation of immune complexes and favouring the binding of IgG to activated FcγR (Fc gamma receptor)^39–41^. Moreover, Fc-linked glycans appear to modulate the activation of complement system. Whereas the classical rout of complement can be triggered by the preferential binding of C1q to fully glycosylated IgG, the lectin pathway is recruited through the recognition of agalactosylated IgG by mannose-binding lectin^42^. In opposite, the presence of terminal galactose and/or sialic acid residues on Fc glycans can be linked with anti-inflammatory properties of IgG^41^. Thus, variations in the glycan structures of IgG Fc can switch the immune system toward a pro- to anti-inflammatory response, by modulating the interaction of IgG with several immune components, including FcγR, complement factors and lectins^43^.

We observed reduced IgG galactosylation in non-endometriosis women, when compared to the control group, which is probably due to the fact that NE group involved patients treated and operated for benign gynaecological conditions and some of these pathologies, e.g. tubal infertility, uterine fibroids or ovarian cysts may be accompanied by inflammation. Some studies reported that changes in IgG galactosylation are also observed in physiological conditions and it is known that they are age^44,45^ and sex^46^ related. In our study, we did not observe any correlations between changes in the level of IgG galactosylation and the age of patients in any of the studied groups (data not shown). Decreased levels of terminal galactose on biantennary IgG glycans may indicate that the activity of galactosyltransferases responsible for decorating N-glycans with galactose residues is impeded^25^. Glycoforms lacking terminal galactose are particularly pro-inflammatory^39,41,47^, while galactosylation strongly decreases pro-inflammatory function of IgG^41^. This is confirmed by results of our research, in which E and NE groups significantly lower reactivities of s-IgG and i-IgG with a specific lectins, compared to the healthy women, were observed. Another observation we made, is that non-endometriosis group, with benign gynecological diseases should not be used as a representative control for differentiation with endometriosis, because IgG glycosylation varies with inflammation, which can accompany a wide variety of diseases, not endometriosis only.

In our study the reaction of reduced s-IgG and i-IgG with terminal galactose-specific RCA-I and terminal GlcNAc-specific GSL-II was reciprocal to each other, because terminal GlcNAc residues are exposed in glycoprotein oligosaccharides after galactose removal. These reactions were not always exactly reciprocal, what may be explained in two ways. First of them is more general and is based on the fact that measurements using the lectins it is not in practice the total amount of a specific glycotope present on a protein, but rather the amount of this glycotope currently accessible for the lectin. Unfolding of a polypeptide chain, after IgG reduction, strongly influences this feature. Secondly, it should be taken into account that some oligosaccharide chains may be present statistically also in Fab fragment of IgG^48^. Like galactose requires the terminal GlcNAc as a substrate, sialic acid requires galactose moiety^41^. Increased sialylation of IgG generally results from the increased galactosylation, because galactosylated IgG is the substrate for sialyltransferases^49^. On human glycoproteins, sialic acids may be bound α2,3 or α2,6 to galactoses and are the most exposed monosaccharides to the outer environment, participating in many biological processes including *inter alia* cancerogenesis^50^. In healthy adults mono- and disialylated glycan structures of IgG represent about 10–15% of total IgG Fc oligosaccharide structures^22^. Terminal sialic acids appear to mainly serve as a switch between IgG pro- and anti-inflammatory activity in cases of homeostasis disturbance, what is in contrast to galactosylation, which seems to represent an interface between physiological and pathological processes. This is confirmed by the results of our research, in which we observed a higher reactivity of IgG with sialic acid-specific lectins in the control group. This is in accordance with the results of Maignien et al.^51^, who reported reduced α2,6 sialylation in the peritoneal fluid of women with endometriosis. Authors documented that the IgG reactivity with SNA in the endometriosis group was significantly lower when compared to the control group. In contrast, they did not observe any difference in the expression of sialic acid α2,3-linked. Authors concluded, that the mechanisms of altered sialylation during endometriosis development, especially those affecting the adhesion between endometrial cells and peritoneal mesothelial cells, are still unclear^16^. It has been reported that the glycoproteins levels are increased in peritoneal fluid, serum, and eutopic endometrium of women with endometriosis^38,52^. However, in our previous study we documented, that IgG concentration was significantly lower in severe endometriosis than in the control group of healthy women^53^.

In our study, we showed a strong positive correlation between IgG RCA-I reactivity and its reactivity with lectins specific for sialic acid. RCA-I has a preference for a Galβ1,4 rather than Galβ1,3 or Galβ1,6 terminal sequences. However, there seems to be no specific requirement for the sub‐terminal residue, e.g. Galβ1,4GlcNAc, Galβ1,4Glc and Galβ1,4Man have shown similar activities. The presence of sialic acid at the 3-O or 6-O positions modifies the terminal Gal, significantly reducing the binding affinity to RCA-I^54^. Structurally, the N-linked glycans of human IgGs are typically biantennary chains. The heterogeneous IgG glycans can be classified into three sets (G0, G1, and G2), depending on the number of galactose residues in the outer arms of biantennary glycans^55^.

Comparing the lectins relative reactivity of s-IgG and i-IgG glycans in each of the analysed groups, they are more or less different from each other, what may be due to differences in the availability of sugar residues for lectins between native s-IgG and i-IgG isolated from serum. On the other hand the significant correlations between s-IgG and i-IgG reactivity with lectins used were observed (the highest for MAA and the lowest for RCA-I). Analysing the results obtained and the utility of them to differentiate serum samples in a way that reflects their clinical characteristics typical for endometriosis, we observed, that the values of agalactosylation factor and sialylation factor are much better than the values of relative reactivities of IgG glycans with each lectin separately, with one exclusion—MAA relative reactivity with sialic acid α2,3-linked. The above observation is in agreement with results of ROC analysis in which both for s-IgG and i-IgG cut-off point for MAA reactivity was 0.014 AU (AUC 0.812) and 0.020 AU (AUC 0.970), respectively, for MAA/SNA factor it was 0.039 (AUC 0.762) and 0.027 (AUC 0.916), respectively, and for AF it was 0.063 (AUC 0.800) and 0.055 (AUC 0.886). Although the results of the ROC analysis showed that for i-IgG also the other parameters may be taken into account as markers of endometriosis (except the value of i-IgG relative reactivity with GSL-II), we selected only three of them for the cluster analysis, those which for s-IgG had a moderate AUC value. The results of cluster analysis additionally confirm the utility of the set of three selected parameters: MAA relative reactivity, MAA/SNA ratio and agalactosylation factor values for advanced endometriosis differentiation from group of healthy women. For s-IgG of the four clusters formed, cluster 3 differ with regard to the clinical characteristics of women, gathering 74% of samples of healthy women (n = 14) and 9 samples from endometriosis group (22.5%). From 5 clusters formed for i-IgG, cluster 5 gathered 63% of samples from the control group (n = 12) and 2.5% (n = 1) of serum samples from women with endometriosis. Taking into account the thesis of our investigations, the aim of which was to check whether the analysis of sialylation and galactosylation/agalactosylation degree of s-IgG glycans, without the prior time-consuming, complicated procedure of protein isolation, may be usable as differentiating marker/markers for endometriosis, our results seem to meet this criterion. It should also be mentioned that in lectin-ELISA method, glycan-lectin reaction reflect the reactions that occur in living organisms, including the availability of sugar residues for lectins, which additionally allows to deepen the knowledge about mechanisms of these interactions.

## Materials and methods

Serum samples from women with diagnosed III and IV stages of endometriosis (E; n = 40, the median age: 34 years [interquartile range (IQR) 30.5–40.5]) as well as from group of women without endometriosis (NE—non-endometriosis; n = 36, the median age: 39 years [IQR 33.5–42.0]), were collected at the Department of Oncological Gynecology, Wroclaw Comprehensive Cancer Center, Poland. The study was conducted in agreement with the Helsinki-II-declaration and the protocol was approved by the Bioethics Human Research Committee of the Wroclaw Medical University (Permission No. KB-293/2016 and KB-719/2018). E and NE groups underwent surgical interventions, mainly laparoscopic, and after histological verification were classified to the proper group. Endometriosis women were classified on extend and severity of disease according to the revised American Fertility Society (rAFS) classification. Serum samples from healthy women (C—control group, n = 19, the median age: 39 years [IQR 35.0–48.0]), were collected at the Department of Laboratory Diagnostics, Wroclaw Medical University (positive opinion of Bioethics Committee No KB-117/2020). Non-endometriosis group was histologically confirmed with benign ovarian cyst, with severe dysplasia—CIN 3 (cervical intraepithelial neoplasia grade 3) or leiomyoma’s. The control group was consisted of healthy women, with no symptoms or history connected with endometriosis, non-pregnant, without any gynecological diseases. Before starting the study, all participants gave a written and informed consent.

The profile and degree of IgG sialylation and galactosylation/agalactosylation was determined using modified solid phase enzyme-linked immunosorbent assay, lectin-ELISA described by us earlier^31–34^. The method was based on the reactivity of IgG glycan moieties with specific biotinylated lectins: *Maackia amurensis* agglutinin (MAA, recognizing sialic acid α2,3-linked) and *Sambucus nigra* agglutinin (SNA, which recognize sialic acid α2,6-linked), *Ricinus communis agglutinin I* (RCA-I) which detect the terminal galactose; *Griffonia simplicifolia* lectin II (GSL-II) detecting the terminal GlcNAc (Vector Laboratories Inc., Burlingame, CA, USA).

### Lectin-ELISA

The microtiter plates were incubated with 0.01 mg/ml protein G (abcam, USA) solution in 10 mM TBS pH 7.4 for 2 h at 37 °C, next, 4 °C overnight. Then the plates were coated with native/isolated IgG diluted 10 mM TBS-T (TBS containing 0.1% Tween, pH 7.4) in an amount 500 ng IgG in 50 µl solution per well, and incubated for 3 h at 37 °C. After the incubation, the reduction with dithiothreitol (DTT) for 70 min at 37 °C was carried out. Then the plates were incubated with biotinylated lectins (90 min, 37 °C) diluted with 10 mM TBS-T as follows: MAA: 1:250, SNA: 1:2000, RCA-I: 1:500, GSL-II: 1:400. Next, the plates were incubated with phosphatase-labelled ExtrAvidin for 1 h at room temperature. After incubation the phosphatase reaction was developed with p-nitrophenyl-phosphate used as a substrate (37 °C). The reaction was stopped with 100 µl of 1 mM NaOH per well and the absorbance was measured at 405 nm, reference filter 630 nm, with Mindray-96A microplate reader. After each incubation step the plates were extensively washed with 10 mM TBS-T, pH 7.4. All samples were analysed in duplicate. Background absorbance was measured for samples in which all reagents were present, but biological sample was replaced with 10 mM TBS-T. Samples relative reactivities with lectins were expressed in absorbance units (AU). IgG concentration values in whole sera (s-IgG), necessary for calculation of IgG amount to lectin-ELISA, were estimated by us previously using turbidymetric method^53^ and those in IgG isolates (i-IgG) were measured by BCA method (see below).

### IgG isolation

Immunoglobulin G was isolated from serum samples using affinity chromatography on Protein A/Protein G Sepharose column, according to the procedure described earlier by Ey et al.^56^. The serum sample (0.5 mL) was diluted 1:1 with 50 mM TBS, pH 8.0, applied on the column (1 mL) and washed using starting TBS solution. IgG, retained on the column, was eluted with 0.1 M glycine/HCl, pH 2.7 and immediately neutralized with 1 M Tris to avoid IgG degradation. Elution profile was determined by measuring the absorbance at 280 nm. The fractions containing IgG were pooled, concentrated using Amicon Ultra-15 centrifugal filter unit with ultracel-100 membrane (Millipore). IgG concentration was determined spectrophotometrically on a polystyrene 96-well microtiter plate (Maxisorp, Dako, Denmark) using bicinchoninic acid (BCA) colorimetric micromethod^57^. Briefly, to the 10 μL of IgG solution, diluted with water if necessary, an aliquot of 200 μL mixture of stock A and B solutions, in the proportion 50:1, was added and the plate was incubated at 37 °C for 30 min, as a standard the bovine serum albumin (BSA) in the range 0–10 μg/well was used. The absorbances were measured at 562 nm against blank, and the protein concentrations were read from the standard curve and expressed in μg/μl.

### Statistical analysis

Statistical analysis was performed using the statistical software STATISTICA 13.3PL (StatSoft). Experimental data were presented as means and standard deviations (SD), and distribution of the values within analysed groups was presented as box-whisker plots with median and interquartile (25th–75th percentile) range. According to a Shapiro–Wilk *W* test, the values did not fit normal distribution, thus the nonparametric Mann–Whitney *U* test was used to determine differences among the groups. The correlations with 95% of confidence interval were estimated according to the Spearman rank test. A two-tailed *p*-value of less than 0.05 was considered as significant. The diagnostic significance of determined parameters was analysed using receiver operating characteristic (ROC) curves. Moreover, cluster analysis was performed for glycans expression on serum native IgG and isolated IgG, only for those parameters for which the AUC values, determined in ROC analysis, simultaneously were moderate or high. In this analysis, the results are presented as a dendrogram, starting from one cluster in which all subjects (patients and controls) are gathered. Next, the subjects were clustered, with the similar in terms of the values of all analysed traits grouped together and different ones forming a separate cluster. In general, the greater distance of separation, the greater differences in subject characteristics. The similarities between samples were calculated using an Euclidean metric on the original data points, with no reference to the clinical status of the samples. The scheme of statistical analysis of results obtained in the present study we have adopted is based on our previous experience^53^.

### Ethics approval

The study procedures followed in the study were conducted in agreement with the Helsinki-II-declaration and the protocol was approved by the Bioethics Human Research Committee of the Wroclaw Medical University (Permission No. KB-293/2016 and KB-719/2018). Written informed consent was obtained from recruited patients.

## Conclusion

Endometriosis diagnostics is difficult and often invasive, therefore non-invasive diagnostics methods and parameters are needed. Disease development is associated with inflammatory processes, especially in advanced stages of endometriosis, markers of which can be also detected in peripheral blood serum. In the light of above information, the analysis of glycosylation profile and degree one of those markers, IgG, may be very promising in this context. Thus, the analysis of IgG sialylation and galactosylation/agalactosylation degree could be helpful during advanced endometriosis diagnostics, and may be used as supplementary parameters for medical interview and tests. The proposed panel of parameters, the expression of MAA-reactive sialic acid α2,3-linked, values of MAA/SNA ratio and agalactosylation factor, could be taken into account as a useful clinical tool to elucidate women with high risk of endometriosis development. However, further studies are needed to evaluate its clinical utility as a panel of additional markers for endometriosis diagnostics.

### Limitation of the study

1. Lack of representative early-stage endometriosis group, what makes impossible the verification of parameters analyzed by us and their ability to evaluate as useful biomarkers for early stage of endometriosis.
2. Lack of peritoneal fluid to compare the results of IgG glycosylation analysis with those obtained for serum IgG.

### Strength of the study

1. The panel of IgG glycosylation markers proposed by us (the expression of MAA-reactive sialic acid α2,3-linked, values of MAA/SNA ratio and agalactosylation factor) may be helpful in differentiation and diagnostics of advanced stage of endometriosis.
2. The analyzed by us IgG glycosylation markers could be used as a clinical tool to differentiate women with high risk of severe endometriosis development, qualified for laparoscopy procedure.
3. We showed that the analysis of sialylation and galactosylation/agalactosylation degree of s-IgG glycans, without the prior time-consuming, complicated procedure of protein isolation, may be usable as differentiating markers for advanced endometriosis.
4. The results of our studies can be used as the basis for further studies aimed at searching for noninvasive diagnostic parameters, with potential utility in women with advanced endometriosis.
5. Our study showed that non-endometriosis group, gathering women suffering from benign gynecological diseases with accompanying inflammation, seems not to be a proper comparative group to women with advanced endometriosis, because changes in IgG glycans expression are associated with the development of inflammation. Therefore in our study as a control group we used serum samples obtained from healthy women.

## Supplementary Information

Supplementary Information 1.

## Data Availability

The data that support the findings of this study are available from the corresponding author upon reasonable request.
