# Supplementary material for: Variability of serum IgG sialylation and galactosylation degree in women with advanced endometriosis
Source: Sci Rep. 2021 Mar 10;11:5586. doi: 10.1038/s41598-021-85200-x (PMC7970930; doi:10.1038/s41598-021-85200-x)
Supplement: Supplementary file 1 — Supplementary Information 1. [file 41598_2021_85200_MOESM1_ESM.docx]

**Supplementary materials**

**Variability of serum IgG sialylation and galactosylation degree in women with advanced endometriosis**

Katarzyna Sołkiewicz, Hubert Krotkiewski, Marcin Jędryka, Ewa M. Kratz

|  |  |
| --- | --- |

**Fig. 1S. Correlations between relative reactivities of IgG glycans with lectins (a-r).** Serum native IgG (s-IgG); isolated IgG (i-IgG). The correlations were estimated according to Spearman test and a two tailed *p*-value of less than 0.05 was considered as significant. The dashed line shows 95% of confidence interval. For lectins specificity see Materials and methods section.

**Fig. 2S. Correlations between relative reactivities of specific lectins with s-IgG and i-IgG glycans (a-f).** Serum native IgG (s-IgG); isolated IgG (i-IgG). The correlations were estimated according to Spearman test and a two tailed *p*-value of less than 0.05 was considered as significant. The dashed line shows 95% of confidence interval. For lectins specificity see Materials and methods section.

**Fig. 3S. ROC curve analysis of serum native IgG (s) and isolated serum IgG (i) relative reactivity with lectins.** MAA (*Maackia amurensis* agglutinin), SNA (*Sambucus nigra* agglutinin), RCA-I (*Ricinus communis* agglutinin I), GSL-II (*Griffonia simplicifolia* lectin II) and MAA/SNA ratio, GSL-II/RCA-I ratio. The analysis was done for patients with advanced endometriosis and in control group of healthy women. For lectins specificity see Materials and methods section.

**Tab. 1S. ROC curve analysis of serum native IgG (s) and isolated serum IgG (i) relative reactivity with lectins**

| **Lectins/**  **lectins reactivity ratio** | **AUC** | **AUC with 95%**  **Confidence interval** | **Cut off point** | **Sensivity** | **Specificity** | **p** |
| --- | --- | --- | --- | --- | --- | --- |
| MAA (s) **^3Sa^** | **0.812** | 0.705 - 0.918 | 0.014 | 0.795 | 0.795 | 0.0000 |
| SNA (s) **^3Sc^** | 0.575 | 0.428 - 0.722 | 0.242 | 0.364 | 0.842 | 0.3151 |
| MAA/SNA (s) **^3Se^** | **0.762** | 0.644 - 0.880 | 0.039 | 0.659 | 0.895 | 0.0000 |
| RCA-I (s) **^3Sg^** | 0.699 | 0.559- 0.838 | 0.424 | 0.864 | 0.474 | 0.0053 |
| GSL-II (s) **^3Si^** | 0.675 | 0.520 - 0.830 | 0.016 | 0.866 | 0.474 | 0.0272 |
| GSL-II/RCA-I (s) **^3Sk^** | **0.800** | 0.676 - 0.924 | 0.063 | 0.750 | 0.842 | 0.0000 |
| MAA (i) **^3Sb^** | **0.970** | 0.924 -1 | 0.020 | 0.886 | 1.000 | 0.0000 |
| SNA (i) **^3Sd^** | 0.943 | 0.887 - 0.998 | 0.352 | 0.864 | 0.947 | 0.0000 |
| MAA/SNA (i) **^3Sf^** | **0.916** | 0.847 - 0.986 | 0.027 | 0.773 | 1.000 | 0.0000 |
| RCA-I (i) **^3Sh^** | 0.922 | 0.856 - 0.988 | 0.365 | 0.795 | 1.000 | 0.0000 |
| GSL-II (i) **^3Sj^** | 0.539 | 0.393 - 0.686 | 0.030 | 0.455 | 0.737 | 0.5976 |
| GSL-II/RCA-I (i) **^3Sl^** | **0.886** | 0.801 - 0.972 | 0.055 | 0.864 | 0.842 | 0.0000 |

MAA (*Maackia amurensis* agglutinin), SNA (*Sambucus nigra* agglutinin), RCA-I (*Ricinus communis* agglutinin I), GSL-II (*Griffonia simplicifolia* lectin II) and MAA/SNA ratio, GSL-II/RCA-I ratio in advanced endometriosis and control group of healthy women. The analysis was done for patients with advanced endometriosis and in control group of healthy women. For lectins specificity see Materials and methods section. 3Sa-3Sl – see Figure 3S.

**Fig. 4S. Dendrogram of cluster analysis for values of s-IgG MAA relative reactivity, MAA/SNA ratio and GSL-II/RCA-I factor.** E – endometriosis, C – control group. The cluster analysis was done only for parameters for which in ROC analysis the AUC value was moderate (≥0.762). Each serum sample is represented by a vector of three features: MAA relative reactivity, MAA/SNA ratio, GSL-II/RCA-I factor.

**Tab. 2S. Results of cluster analysis for values of s-IgG MAA relative reactivity, MAA/SNA ratio and GSL-II/RCA-I factor**

| **Group**  **Cluster No** | **C**  **N=19** | **E**  **N=40** |
| --- | --- | --- |
|  | The number  (percentage participation in whole group) | |
| **4** | **2**  (10 %) | **19**  (47.5%) |
| **3** | **14**  (74 %) | **9**  (22.5%) |
| **2** | **3**  (16%) | **9**  (22.5%) |
| **1** | **0**  (0%) | **3**  (7.5%) |

E – endometriosis, C – control group. The cluster analysis was done only for parameters for which in ROC analysis the AUC value was moderate (≥0.762). Each serum sample is represented by a vector of three features: MAA relative reactivity, MAA/SNA ratio, GSL-II/RCA-I factor.

**Fig. 5S. Dendrogram of cluster analysis for values of i-IgG MAA relative reactivity, MAA/SNA ratio and GSL-II/RCA-I factor.** E – endometriosis, C – control group. The cluster analysis was done only for parameters for which in ROC analysis the AUC value was moderate (≥0.762). Each serum sample is represented by a vector of three features: MAA relative reactivity, MAA/SNA ratio, GSL-II/RCA-I factor.

**Tab. 3S. Results of cluster analysis for values of i-IgG MAA relative reactivity, MAA/SNA ratio and GSL-II/RCA-I factor**

| **Group**  **Cluster No** | **C**  **N=19** | **E**  **N=40** |
| --- | --- | --- |
|  | The number  (percentage participation in whole group) | |
| **5** | **12**  (63%) | **1**  (2.5%) |
| **4** | **3**  (15%) | **24**  (60%) |
| **3** | **0**  (0%) | **13**  (32.5%) |
| **2** | **4**  (21%) | **0**  (0%) |
| **1** | **0**  (0%) | **2**  (5%) |

E – endometriosis, C – control group. The cluster analysis was done only for parameters for which in ROC analysis the AUC value was moderate (≥0.762). Each serum sample is represented by a vector of three features: MAA relative reactivity, MAA/SNA ratio, GSL-II/RCA-I factor.
